# Proteasomal degradation competes with Mia40-mediated import into mitochondria

**DOI:** 10.1186/s12915-018-0537-0

**Published:** 2018-06-22

**Authors:** Eva Zöller, R. Todd Alexander, Johannes M. Herrmann

**Affiliations:** 1 Cell Biology, University of Kaiserslautern, Erwin-Schrödinger-Strasse 13, 67663 Kaiserslautern, Germany; 2 Department of Pediatrics, University of Alberta, Edmonton, AB T6G 1C9 Canada

## Abstract

Tandem fluorescent protein timers are elegant tools to determine proteolytic stabilities of cytosolic proteins with high spatial and temporal resolution. In a new study published in *BMC Biology*, Kowalski et al. fused timers to precursors of proteins of the mitochondrial intermembrane space and found that they are under surveillance of the ubiquitin-proteasome system. Ubiquitination at lysine residues of these precursors directly inhibits their translocation into the intermembrane space and targets them for proteasomal degradation.

## Commentary

Mitochondria are essential organelles of eukaryotic cells that contain 800 to 1500 different proteins. Recent comprehensive proteomic approaches provide a fascinating detailed picture of the complement and abundance of essentially all mitochondrial proteins [1]. With the exception of a few mitochondrially encoded proteins, mitochondrial proteins are synthesized in the cytosol as precursors, necessitating that they be imported through translocases in the outer and inner membranes of the mitochondria, called TOM and TIM complexes, respectively. Proteins of the intermembrane space (IMS) use a specific import mechanism that relies on the IMS protein Mia40. Mia40 is an oxidoreductase which introduces disulfide bonds into its substrates in a reaction that occurs simultaneously to their translocation across the outer membrane [2]. The components and mechanisms that drive the import of mitochondrial proteins have been characterized, predominantly by the use of very powerful in vitro assays. However, due to the limitations of such assays, our knowledge of the dynamics and timing of these reactions is poor. It is unclear how long it takes for a mitochondrial precursor produced by a cytosolic ribosome to reach the surface of the mitochondria, to be threaded through the translocase(s), and to fold and assemble into their native states. Mitochondrial precursor proteins do not accumulate significantly in the cytosol, even if mitochondrial import is inhibited. This implies that they are rapidly degraded by proteolysis [3]. However, again, to what extent and how rapidly this degradation occurs in vivo is not known and are both challenging and important questions in the field of mitochondrial biogenesis.

### Tandem fluorescent protein timers are powerful reporters to assess precursor stability in vivo

**Fig. 1:**
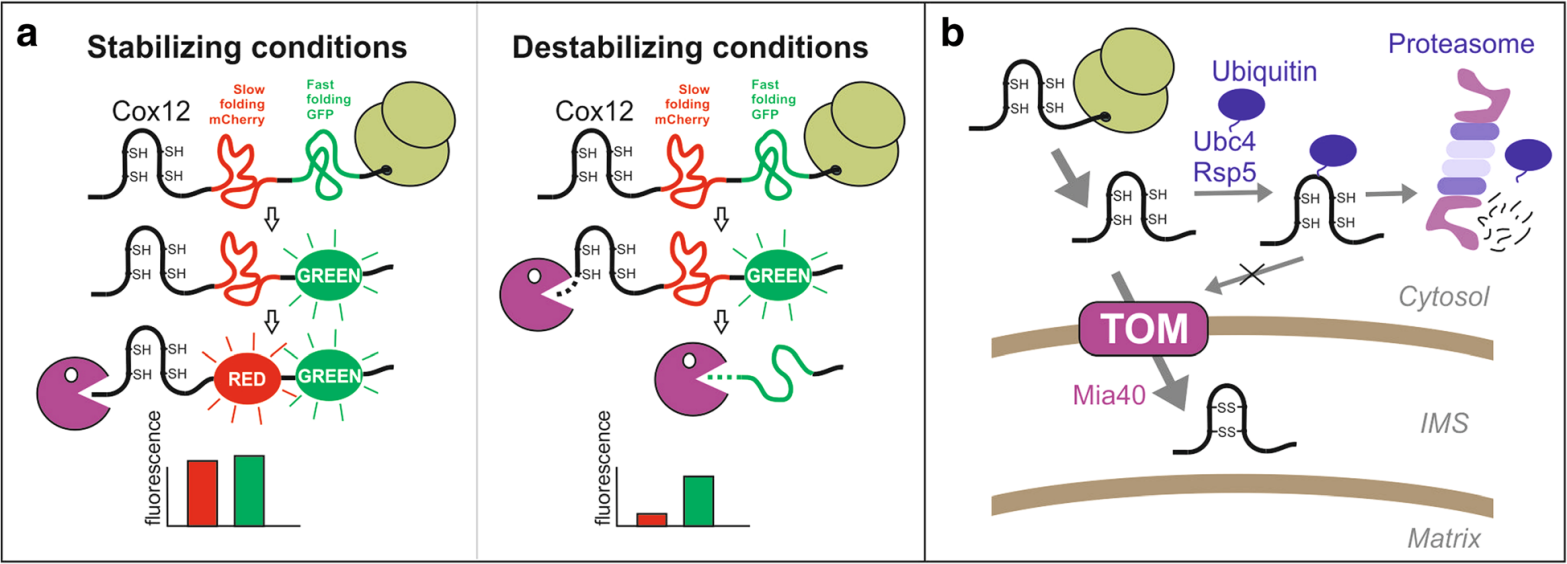
Tandem fluorescent protein timer (tfT) approach to measure cytosolic protein turnover by the ubiquitin-proteasome system. **a** IMS proteins are tagged with a slow maturating mCherry and a fast maturating superfolder green fluorescent protein (sfGFP). The ratio of red (mCherry) to green (sfGFP) intensities correlates with protein stability. **b** Protein import of Mia40 substrates into the IMS competes with their ubiquitin-dependent degradation in the cytosol

The recent study published by Kowalski et al. in *BMC Biology* elucidated whether and how mitochondrial precursor proteins are degraded in the cytosol before they are imported into mitochondria using an innovative strategy [4]. They used a tandem fluorescent protein timer (tFT), an elegant screening tool to assess the stabilities of cytosolic proteins in vivo (Fig. 1). The tFT timer consists of two fluorescent proteins, a slow folding mCherry domain and superfolder GFP (sfGFP). Measuring the fluorescent intensity of both fluorescent proteins and forming a ratio of mCherry/sfGFP makes it possible to determine the degradation speed of the fusion proteins (Fig. 1a). Since unstable proteins are largely degraded before mCherry has time to fold, the ratio of mCherry/sfGFP increases with increasing stability of the fusion proteins [5].

Kowalski et al. attached these timers to sequences of IMS proteins and expressed them in yeast [4]. Since the large fluorescent protein domains prevented protein translocation into mitochondria, these fusions were thereby trapped in the cytosol. Fusion constructs with different IMS proteins strongly differed in their stability. Among the six Mia40 substrates tested, Cox12, a helix-loop-helix protein associated with cytochrome *c* oxidase, was found to be the most destabilizing sequence.

### Cytosolic precursors of IMS proteins are degraded by the proteasome

Using the Cox12-tFT fusion protein as a model protein, they established an assay to study its cytosolic degradation. The activity of the proteasome was found to be essential for the degradation of Cox12-tFT, as well as the presence of lysine residues in the Cox12 sequence that are critical for the attachment of ubiquitin. Particularly mono-ubiquitinated Cox12 was observed in cells, either because poly-ubiquitinated Cox12 is barely formed or because it is rapidly degraded [6]. Thus, IMS proteins are clearly under surveillance of the cytosolic ubiquitin-proteasome system, and different Mia40 substrates show a large variability in respect to their degradation probabilities.

The Cox12-tFT fusion protein allowed the authors to systematically screen for mutants in which this reporter was stabilized. Many proteasome mutants exhibited an increased stability of this reporter, confirming the role of the proteasome in Cox12 degradation. Interestingly, the E2 ubiquitin-conjugating enzyme Ubc4 and the E3 ubiquitin ligase Rsp5 were also found to be critical for the destabilization of the Cox12-tFT reporter. Ubc4 promotes mono- and poly-ubiquitination and is strongly increased upon heat stress in order to stimulate degradation of misfolded proteins. Ubc4 was reported to be critical for the degradation of cytosolic precursors of cytochrome *c*, the most abundant IMS protein [7], and presumably promotes the degradation of a broad spectrum of proteins. Rsp5 is an essential E3 enzyme that promotes both mono- and poly-ubiquitination of targets. It is involved in many processes, including endocytosis and lipid biosynthesis. Interestingly, Rsp5 is critical for the ubiquitination of subunits of the ERMES complex, which connects mitochondria to the endoplasmic reticulum (ER). Rsp5-mediated ubiquitination of the ERMES subunits Mdm34 and Mdm12 represents a critical step in the induction of mitophagy [8].

### Cytosolic ubiquitination of IMS precursors competes with their mitochondrial import

Mitochondrial proteins, regardless of whether they are targeted to the matrix or the IMS, are imported as unfolded polypeptide chains. Tightly folded protein domains, such as that of dihydrofolate reductase or titin, prevent efficient translocation. Though smaller than these domains, the covalent binding of the 9-kDa protein ubiquitin also blocks translocation of the model protein Cox12 [4]. Thus, the ubiquitination machinery in the cytosol appears to compete with mitochondrial import machinery (Fig. 1b). Non-productive import intermediates whose full translocation is blocked due to binding of ubiquitin obviously need to be removed from the TOM complex in the outer membrane. A recent study suggested that the ATP-hydrolysing protein Msp1 recognizes stalled intermediates to pass them on to the proteasome [9]. The accumulation of mitochondrial precursors in the cytosol, for example, due to partial inactivation of Mia40, leads to the induction and/or activation of the proteasome in order to rapidly remove mitochondrial precursors from the cytosol [10]. It will be very interesting in the future to better understand the role of the cellular quality control system for mitochondrial biogenesis. The components and mechanisms of ER-associated degradation (ERAD) have been studied in detail for many years. It seems likely that the ubiquitin-proteasome system has a similarly tight functional and structural association with mitochondria as with the ER, the discovery of which will be an exciting and emerging new avenue in cell biological research.
